# Protein phosphatase complement in rice: genome-wide identification and transcriptional analysis under abiotic stress conditions and reproductive development

**DOI:** 10.1186/1471-2164-11-435

**Published:** 2010-07-16

**Authors:** Amarjeet Singh, Jitender Giri, Sanjay Kapoor, Akhilesh K Tyagi, Girdhar K Pandey

**Affiliations:** 1 Department of Plant Molecular Biology, University of Delhi South Campus, Benito Juarez Road, New Delhi-110021, India; 2 National Institute of Plant Genome Research, Aruna Asaf Ali Marg, New Delhi-110067, India

## Abstract

**Background:**

Protein phosphatases are the key components of a number of signaling pathways where they modulate various cellular responses. In plants, protein phosphatases constitute a large gene family and are reportedly involved in the regulation of abiotic stress responses and plant development. Recently, the whole complement of protein phosphatases has been identified in *Arabidopsis *genome. While PP2C class of serine/threonine phosphatases has been explored in rice, the whole complement of this gene family is yet to be reported.

**Results:**

*In silico *investigation revealed the presence of 132-protein phosphatase-coding genes in rice genome. Domain analysis and phylogenetic studies of evolutionary relationship categorized these genes into PP2A, PP2C, PTP, DSP and LMWP classes. PP2C class represents a major proportion of this gene family with 90 members. Chromosomal localization revealed their distribution on all the 12 chromosomes, with 42 genes being present on segmentally duplicated regions and 10 genes on tandemly duplicated regions of chromosomes. The expression profiles of 128 genes under salinity, cold and drought stress conditions, 11 reproductive developmental (panicle and seed) stages along with three stages of vegetative development were analyzed using microarray expression data. 46 genes were found to be differentially expressing in 3 abiotic stresses out of which 31 were up-regulated and 15 exhibited down-regulation. A total of 82 genes were found to be differentially expressing in different developmental stages. An overlapping expression pattern was found for abiotic stresses and reproductive development, wherein 8 genes were up-regulated and 7 down-regulated. Expression pattern of the 13 selected genes was validated employing real time PCR, and it was found to be in accordance with the microarray expression data for most of the genes.

**Conclusions:**

Exploration of protein phosphatase gene family in rice has resulted in the identification of 132 members, which can be further divided into different classes phylogenetically. Expression profiling and analysis indicate the involvement of this large gene family in a number of signaling pathways triggered by abiotic stresses and their possible role in plant development. Our study will provide the platform from where; the expression pattern information can be transformed into molecular, cellular and biochemical characterization of members belonging to this gene family.

## Background

Plants constantly encounter a number of abiotic stresses such as drought, cold, salinity, osmotic stress in the environment. Plants have evolved complex molecular mechanisms by which they adapt and tolerate these adverse growth conditions. When they perceive stress conditions, plant cells reprogram their cellular processes by triggering a network of signaling events leading to changes in gene expression and eventually altered cellular response. In the post-genomic era, the complete genome sequences of a number of plant species have led to the identification of diverse gene families involved in abiotic stress responses and have unveiled the presence of intricate machinery that leads to the development of tolerance or adaptation against adverse conditions. Many signaling components such as second messengers, sensor-relay, sensor-responders, and effectors and finally the target proteins such as transcription factors, transporters and channel proteins have been implicated in plant stress response.

Reversible protein phosphorylation mediated by protein kinases and protein phosphatases is a major event in signal transduction, regulating many biological processes including cell cycle events, growth factor response, hormone and other environmental stimuli, metabolic control and developmental events [1-6]. During phosphorylation, a protein kinase adds a phosphate group to a substrate. Protein phosphatases reverse this process by removing the phosphate group. In many cases, the addition or removal of a phosphate group to or from an enzyme either activates or deactivates the enzyme effectively. In this manner, protein kinases and phosphatases play a critical role in controlling the activity of an enzyme and, as a result, regulate the biochemical process in which the enzyme participates.

Based on the amino acid residue they preferentially dephosphorylate, protein phosphatases can be categorized into serine/threonine and tyrosine phosphatase. The serine/threonine phosphatases were initially categorized into two groups, PP1 and PP2, based on their substrate specificity and pharmacological properties. PP1s are highly conserved and ubiquitous phosphatases across all eukaryotes. In case of plants, there is only limited knowledge about PP1 so far [7-9]. A PP1 gene up-regulated by biotic stress was reported in *Phaseolus vulgaris *[10]. In *Arabidopsis*, a family of nine PP1 genes has been identified [8,11]. Although functional evidence for these PP1 phosphatases has been difficult to obtain, work with a PP1 phosphatase in *Vicia faba *has demonstrated its involvement in stomata opening in response to the blue light [12]. The PP2 phosphatases have been further subdivided into three classes based on their requirement for divalent cations for the catalysis. PP2A phosphatases do not require divalent cations, while PP2B and PP2C require Ca^2+ ^and Mg^2+ ^, respectively [9]. Based on sequence and structural analysis, type one (PP1), type 2A (PP2A), and type 2B (PP2B) protein phosphatases are related enzymes and hence, are defined as the PPP family. The type 2C protein phosphatases (PP2C), pyruvate dehydrogenase phosphatase and other Mg^2+^-dependent Ser/Thr phosphatases are closely related and share no sequence homology with PPP and thus, form a distinct group, the PPM family [13,14]. Despite their lack of sequence similarity, members of the PPP and PPM families share a similar structural fold [15], suggesting a common mechanism of catalysis. However, even within the same family, significant structural diversity can be generated by the presence of unique regulatory and targeting domains or by the attachment of a regulatory subunit to the catalytic subunit.

Protein tyrosine phosphatases (PTPs) super-family has been classified into tyrosine-specific PTPs that act on phosphotyrosine and dual-specificity protein tyrosine phosphatase (DsPTP), which can dephosphorylate both phosphotyrosine and phosphoserine/phosphothreonine [16,17]. Unique three-dimensional structure of catalytic domain and lack of sequence homology with protein ser/thr phosphatases, indicate that PTPs evolved independently [18]. However, the highly conserved structure of the catalytic domains within the PTP superfamily suggests a common phosphate hydrolysis mechanism [18]. All the members of the PTP superfamily carry the signature motif of CX_5_R in their active site and cysteine is required for PTP catalytic activity [18]. The low molecular weight protein tyr phosphatases (LMW-PTPs), constituting an evolutionarily distinct group, which have converged on a similar catalytic mechanism [19].

Like protein kinases, phosphatases from plants are also expected to perform the pivotal functions in signal transduction network at different developmental stages of plant and under multiple stress conditions. Currently, several research groups are engaged in deciphering the involvement of different kinases such as CDPKs [20], CIPKs [21,22], and MAPKs [23,24] in abiotic and biotic stress signaling networks, in both *Arabidopsis *and rice. Phosphatases are the essential kinase-counteracting component in both eukaryotes and prokaryotes in diverse signaling pathways. Moreover, most of the phosphatases have been studied in *Arabidopsis *and very few have been functionally characterized based on their expression in crop plants. Therefore, it is very crucial to undertake a comprehensive study to understand the role of stress and development regulated phosphatases in rice. In principle, these phosphatases might be the logical candidates for testing an important biological reversible switch of phosphorylation-dephosphorylation in these signaling pathways. Study of the expression pattern of different protein phosphatase classes under various stress conditions and in different plant organs may provide insights into the underlying physiological, biochemical and molecular mechanism of stress tolerance and regulation of development.

In spite of recent identification of the whole complement of protein phosphatases in *Arabidopsis *[25] and genome-wide analysis of PP2C class of phosphatases in both *Arabidopsis *and rice [26], knowledge is minuscule about the expression, structural and functional aspects of protein phosphatases in the regulation of plant growth and development. Also, it is quite obvious that the genome of rice will also comprise phosphatases other than PP2C as found in the genome of *Arabidopsis *[25], which might play very significant role in plant development and stress tolerance. These rationale and availability of the rice genome sequence, online databases and *in silico *search tools enticed us to carry out a detailed analysis towards the identification and expression profiling of protein phosphatases in rice.

In this study, we have identified the full complement of protein phosphatases in rice genome, reporting 132 protein phosphatase-coding genes as well as their structural analysis and expression profiles. We categorized them into different classes by analyzing the catalytic domains they harbor and used phylogenetic analysis to show the relation among the members of various subfamilies. Subsequently, we analyzed the genes for segmental and tandem duplication events, which may have been the likely force for the expansion of this gene family in rice. A detailed expression analysis for *OsPP *(*Oryza sativa *protein phosphatase) genes was done under various environmental stresses as well as during various developmental stages which included vegetative growth, panicle and seed development. This expression analysis will be very useful to envisage the functional role of these genes in abiotic stress signaling, stress tolerance and plant development.

## Methods

### Identification of protein phosphatases in rice genome

The database search was performed using keyword "phosphatase" in RGAP-TIGR (Rice Genome Annotation Project - The Institute of Genomic Research) version 5.0 [27]. This resulted in 321 putative phosphatases, which were then confirmed by the presence of the protein phosphatase domain using SMART (Simple Modular Architecture Research Tool) database [28], using amino acid sequences as query. Out of 321 putative phosphatases, only 118 were found to have the protein phosphatase domain. Keyword search performed on PhosphaBase database [29] fetched 11 new protein phosphatases. Moreover, the TAIR (The Arabidopsis Information Resource) database [30], PhosphaBase, *Saccharomyces *genome database [31] and *Populus *database [32] were mined for *Arabidopsis*, human, yeast and *Populus *genomes, respectively, to extract putative phosphatases. Subsequently, the putative entries were confirmed by the presence of phosphatase domain using SMART database. A common profile was generated from the amino acid sequences of the phosphatase domains of all the 5 organisms (rice, *Arabidopsis*, human, yeast and *Populus*). An HMM (Hidden Markov Model) profile was generated using domains employing HMMER software [33]. This was used as query to search version 5 of RGAP rice pseudomolecules database http://rice.plantbiology.msu.edu/pseudomolecules/info.shtml and the KOME (Knowledge based Molecular Biological Encyclopedia) full-length cDNA database [34] to identify similar sequences, followed by screening for unique entries from two databases. The strategy fetched 141 and 154 unique entries from RGAP and KOME, respectively. All the 141 protein sequences thus obtained from RGAP were again validated for the presence of phosphatase domain employing SMART and InterPro [35] domain analysis tools. Interestingly, 10 out of the 141 were found to be devoid of phosphatase domain, and this led to the identification of 131 protein phosphatases. Protein sequences of 154 unique entries from HMM search in KOME databases were used to BLAST in RGAP rice genome database setting a criterion of ≥ 92% identity. This search resulted in 14 new RGAP locus IDs and these new proteins were also analyzed for the presence of phosphatase domain. Only one out of 14 was found to have phosphatase domain as suggested by InterPro scan.

### Nomenclature and chromosomal localization

Genes are named as *OsPP*x where *Os *indicates *Oryza sativa, PP *indicates protein phosphatase and x is the number assigned to a particular gene (from 1 to 132) in the phosphatase complement. *OsPP *genes were mapped on chromosomes by identifying their positions as given in RGAP database. Information regarding various gene attributes such as ORF size, number of amino acid, number of introns, alternative splicing, expression evidence (cDNA or EST) were collected from RGAP release 5.

### Phylogenetic analysis of OsPPs

Phosphatase domain sequences of rice obtained from SMART database were used for multiple sequence alignment employing ClustalX (version 1.81program) [36]. An un-rooted neighbor-joining (NJ) phylogenetic tree was constructed with the aligned sequences in ClustalX with default parameters. Phylogenetic NJ tree was also made using aligned domain sequences of both, rice and *Arabidopsis *together. Bootstrap analysis was performed using 1000 replicates. The trees thus obtained were viewed using TREEVIEW 1.6.6 software [37].

### Gene duplication

The duplicated genes were found from the RGAP segmental duplication database http://rice.plantbiology.msu.edu/segmental_dup/100kb/segdup_100kb.shtml. Genes separated by 5 or fewer genes were considered tandemly duplicated. The distance between these genes on a chromosome was calculated and the homology in terms of percentage similarity in the amino acid sequences of these gene products was computed employing MegAlign software 5.07^© ^[38].

### Plant material, growth conditions and stress treatment

Tissue at different stages of panicle and seed development was harvested from field-grown rice plants (*Oryza sativa *ssp. *Indica *var. IR64) according to Ray et al. [39]. Collected panicles were frozen in liquid nitrogen immediately after excision to minimize the effect of wounding on individual florets. Treatments for cold, salinity and dehydration stresses to 7-days-old rice seedlings were also given according to Ray et al. [39]. To test the validity of stress treatments given to the seedlings, microarray expression profile was generated (additional file 1 and 2) for few known stress inducible genes [40].

### Microarray based gene expression analysis

Genome wide microarray analysis was performed according to Agarwal et al. [41], to generate the expression profile of *OsPPs. *The samples for the microarray experiment included three vegetative stages (mature leaf, 7 days old seedling and their roots), 11 reproductive stages (P1-P6 and S1-S5; representing panicle and seed developmental stages, respectively) and three abiotic stress conditions, i.e. cold, salt, and dehydration. RNA was isolated from three biological replicates for each stage/treated tissue and microarray experiments were carried out using 51 Affymetrix Gene Chip Rice Genome Arrays (Gene Expression Omnibus, GEO, platform accession number GPL2025) as described. The raw data (*.cel) files generated from all the chips were imported to Array Assist 5.0 software (Stratagene, USA) for detailed analysis. To stabilize the variation of data from all the chips, normalization of the raw data was performed using GC-RMA (Gene Chip Robust Multi-array Analysis) algorithm [42]. Normalized signal intensity values were log transformed and averages of the three biological replicates for each sample were used for further analysis. Student's t-test was performed to identify differentially expressed genes (fold change > 2, at P-value ≤ 0.05) with respect to all vegetative stages in the case of reproductive development and 7 days old unstressed seedling in the case of stress samples. The up- or down-regulated genes in any tissue were calculated from the average of log of normalized signal values. The data for only one probe set per gene (generally 3' most) was used for the analysis. The expression of a particular gene was considered absent if the normalized signal value from the corresponding probe set was < 7. The data was base line transformed by taking the mature leaf and the seedling as the base lines for reproductive stages and stress samples, respectively. On the basis of expression profiles, genes were grouped by using self-organizing maps (SOM) and distance matrix Euclidian on rows (developmental expression) and both rows and columns (stress expression) with 100 maximum iterations. The microarray expression data have been deposited in the gene expression omnibus (GEO) database at NCBI under the series accession numbers GSE6893 and GSE6901.

### Expression analysis by MPSS

MPSS (massively parallel signature sequence) database [43] was explored to obtain the expression profiles of genes that were not represented on the Affymetrix rice Gene Chip^®^. Data was retrieved from 17 bp signatures from selected libraries. Only those signatures, which were unique to the genome and transcribed from the respective strand of the gene (Classes 1 and 2), included in the analysis. A TPM cut-off of > 3 was set to avoid the background signal. The normalized transcript abundance values per million (TPM) were used to assess the expression profile.

### Real time PCR analysis

To validate the microarray data for a few selected genes showing differential expression pattern under abiotic stress conditions, real time PCR was performed using two biological replicates. Primers were made for all the selected genes preferentially, from 3' end, employing PRIMER EXPRESS (PE Applied Biosystems, USA), with default settings. Each primer was checked using BLAST tool of NCBI for its specificity for the respective gene, and also was confirmed by dissociation curve analysis after the PCR reaction (Additional file 3).

4 μg of DNase treated total RNA was used to synthesize the first strand cDNA in 100 μl of reaction volume using high-capacity cDNA Archive kit (Applied Biosystems, USA). SYBRGreen PCR Master Mix (Applied Biosystems, USA) was used to determine the expression levels for the genes in ABI Prism 7000 Sequence detection System (Applied Biosystems, USA). To normalize the variance among samples, *ACTIN *was used as the endogenous control. Relative expression values were calculated employing ΔΔCt method and normalized the data against the maximum average expression value from microarray.

## Results

### Identification of protein phosphatases in rice genome

Keyword search from RGAP resulted in 321 putative phosphatases, which were narrowed down to 118 after domain analysis. During domain analysis various other domains such as S_TKc (ser/thr kinase catalytic domain), FHA (forkhead associated domain), TPR (tetratricopeptide repeat), EF-hand (calcium binding motif) were found to be present in putative candidates along with the phosphatase catalytic domains, PP2Ac, PP2Cc, PTPc, DSPc, PTP_DSPc and LMWPc (Additional file 4). Keyword search in PhosphaBase revealed 11 additional protein phosphatases. From HMM search in RGAP, we found 141 unique entries. When analyzed for the presence of conserved domains employing SMART and InterPro, we found that 10 of these were devoid of any and/or phosphatase domain, therefore, 131 protein phosphatases were confirmed by this approach. Similar HMM search in KOME database resulted in 154 unique entries. BLAST search in RGAP with the amino acid sequences of these unique entries, resulted in 14 new genes. Domain analysis of these new 14 entries by SMART did not reveal phosphatases domain in any of the genes. However, similar analysis using InterPro showed the presence of PP domain (PTP_DSPc) in one (*OsPP62*) of the 14 genes. Hence, the total number of identified protein phosphatase coding gene is 132.

### Organization of rice protein phosphatase gene family

Protein phosphatase genes extracted by keyword search and HMM profile search were categorized into 5 classes depending on the presence of various domains. PP2C with highest number of members formed a major class of 90 genes. PP2A and DSP comprised of 17 and 23 members, respectively, while PTP and LMWP contained one member each. The intron-exon structure analysis revealed a variation of 0 to 20 introns per gene, with about 70% genes containing at least 4 introns. Expression evidences were available for 91% of the genes in terms of ESTs or full-length cDNAs (Additional file 5). Expression of 97% of the *OsPP *family members could be derived from microarray and MPSS based studies.

### Phylogenetic analysis of protein phosphatase gene family

**Figure 1:**
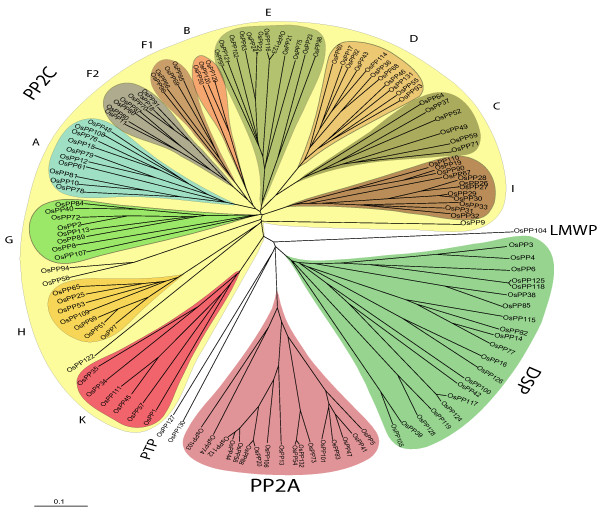
**Phylogenetic relationship among various phosphatase classes of rice**. An un-rooted NJ tree is made from the domains sequences of rice phosphatases. Tree was made using ClustalX 1.81 and viewed in Treeview 1.6.6 software. The whole protein phosphatase gene family is divided into different classes, PP2A, PP2C, DSP, PTP and LMWP, each represented by a clade. PP2C class is further subdivided into different classes (A-K) each represented by a subclade as described by Xue et al [26]. Scale bar represents 0.1 amino acid substitutions per site.

**Figure 2:**
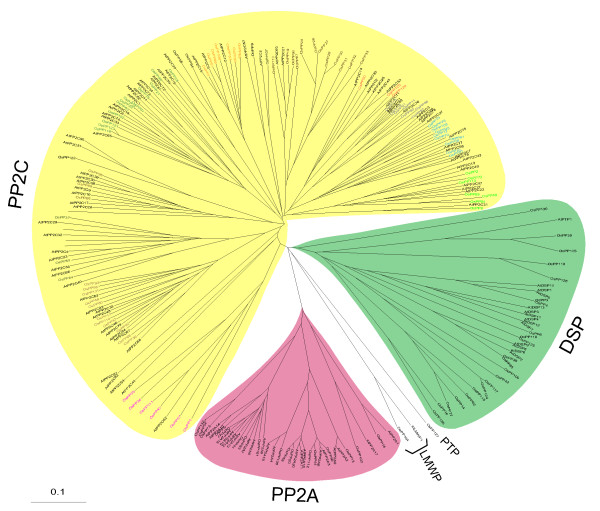
**Phylogenetic analysis of rice and *Arabidopsis *protein phosphatase genes**. An Un-rooted NJ tree made from the domain sequences of rice and *Arabidopsis *protein phosphatases. Tree was made using ClustalX 1.81 and viewed using treeview 1.6.6. software. PPs from rice and *Arabidopsis *belong to same class falling in the same clades are based on the bootstrap support value ≥ 50%. Scale bar represents 0.1 amino acid substitutions per site.

To find out the evolutionary relationship among the members of the protein phosphatase gene family, phylogenetic analysis was carried out based on the catalytic phosphatase domain. All the members of PP2C class of phosphatases formed a single major clade. This clade could be divided into 11 subclades based on ≥ 50% bootstrap support. Each subclade is representing a subfamily of PP2C and is designated from A-K according to Xue et al [26]. PP2A and DSP were two other major classes and formed two separate major clades, with each clade containing all the members of respective classes (domain sequence for *OsPP62 *could not be found and hence not represented in the phylogenetic tree). Single genes belonging to LMWP (*OsPP104*) and PTP (*OsPP127*) classes were positioned separately (Figure 1, Additional file 6). Investigation of the relationship between rice and *Arabidopsis *protein phosphatase gene family revealed very similar tree topologies and subfamily organization to individual rice tree (Figure 2).

### Chromosomal localization and gene duplication

**Figure 3:**
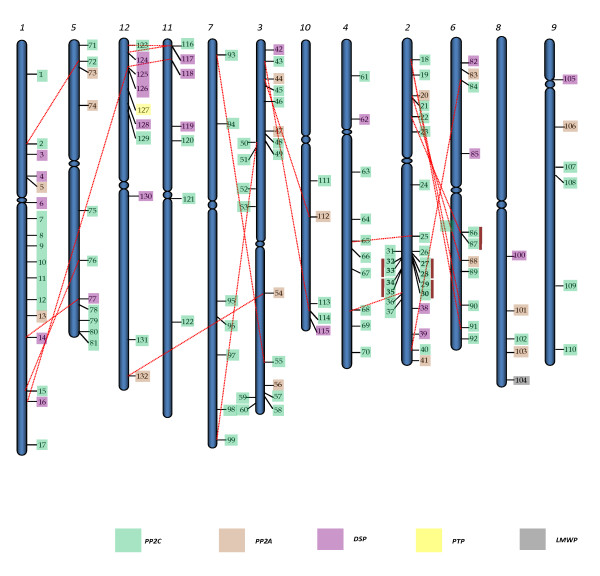
**Chromosomal localization of *OsPP *genes on 12 chromosomes of rice**. Respective chromosome numbers are written at the top. Genes belonging to five classes have been marked by different colors. Corresponding numbers as described in Additionl file 5 indicate gene names. Dashed lines join the genes, lying on duplicated segments of the genome. Tandemly duplicated genes are joined with vertical lines. Chromosomes are grouped randomly to show the duplication with clarity.

The rice PPs were mapped to RGAP pseudomolecules (version 5; chromosome 1-12) based on the coordinates of RGAP loci http://rice.plantbiology.msu.edu/pseudomolecules/info.shtml. Rice protein phosphatases were variably distributed on all chromosomes, with the maximum 24 members located on chromosome 2 and 17 members present on largest chromosome 1 (Figure 3). On the other hand, only 5 genes were localized on chromosome 8 and 10 each. A total of 42 *OsPP *genes were present on segmentally duplicated chromosomal regions (Table 1). Out of these 42 genes, 40 had their counterparts on duplicated segments of the chromosome. On the criterion of separation by less than 5 intervening genes and ≥ 50% homology at protein level, a total of 10 genes were found to be tandemly duplicated, falling into 5 groups with each group comprising of 2 genes (Table 2). All the tandemly duplicated genes were localized only on two chromosomes, 4 pairs on chromosome 2 and one pair on chromosome 6. Categorically, segmentally duplicated genes were found to be distributed as 13 pairs PP2Cs, 4 pairs DSPs, 3 pairs PP2As, whereas all the tandemly duplicated genes were PP2Cs (Figure 3).

**Table 1:** *OsPPs *present in segmental duplication in rice genome

S.N.	Gene ID	Locus ID	S.N.	Duplicated Gene ID	Duplicated locus ID
1	*OsPP2*	LOC_Os01g19130.1	23	*OsPP72*	LOC_Os05g04360.1
2	*OsPP14*	LOC_Os01g53710.1	24	*OsPP77*	LOC_Os05g44910.1
3	*OsPP15*	LOC_Os01g62760.1	25	*OsPP76*	LOC_Os05g38290.2
4	*OsPP16*	LOC_Os01g64010.1	26	*OsPP126*	LOC_Os12g05660.1
5	*OsPP18*	LOC_Os02g05630.1	27	*OsPP91*	LOC_Os06g48300.1
6	*OsPP19*	LOC_Os02g08364.1	28	*OsPP90*	LOC_Os06g44210.1
7	*OsPP20*	LOC_Os02g12580.1	29	*OsPP88*	LOC_Os06g37660.1
8	*OsPP22*	LOC_Os02g15594.1	30	*OsPP87*	LOC_Os06g33549.1
9	*OsPP25*	LOC_Os02g35910.1	31	*OsPP65*	LOC_Os04g37660.1
10	*OsPP36*	LOC_Os02g46080.1	32	*OsPP68*	LOC_Os04g49490.2
11	*OsPP39*	LOC_Os02g53160.1		*	LOC_Os06g10650.1
12	*OsPP40*	LOC_Os02g55560.1	33	*OsPP84*	LOC_Os06g08140.1
13	*OsPP43*	LOC_Os03g04430.1	34	*OsPP114*	LOC_Os10g39780.2
14	*OsPP44*	LOC_Os03g07150.1	35	*OsPP112*	LOC_Os10g27050.1
15	*OsPP49*	LOC_Os03g16760.1	36	*OsPP71*	LOC_Os05g02110.1
16	*OsPP51*	LOC_Os03g18970.1	37	*OsPP99*	LOC_Os07g49040.1
17	*OsPP54*	LOC_Os03g44500.1	38	*OsPP132*	LOC_Os12g42310.1
18	*OsPP55*	LOC_Os03g55320.1	39	*OsPP93*	LOC_Os07g02330.1
19	*OsPP116*	LOC_Os11g01790.1	40	*OsPP123*	LOC_Os12g01770.1
20	*OsPP117*	LOC_Os11g02180.1	41	*OsPP124*	LOC_Os12g02120.1
21	*OsPP118*	LOC_Os11g04180.1	42	*OsPP125*	LOC_Os12g03990.1
22	*OsPP127*	LOC_Os12g07590.1		*	LOC_Os11g07850.1

* Blanks in the gene_Ids in the right hand side column indicate that these TIGR loci were not in the 132 datasets

**Table 2:** *OsPPs *present in tandem duplication in rice genome

Locus ID	Gene	Group	**Distance in kb**^**a**^	**%Homology**^**b**^
LOC_Os02g38690	OsPP27	1	6.55	76.3
LOC_Os02g38710	OsPP28			
LOC_Os02g38780	OsPP29	2	12.26	77.4
LOC_Os02g38804	OsPP30			
LOC_Os02g39470	OsPP32	3	15.81	54.3
LOC_Os02g39480	OsPP33			
LOC_Os02g42250	OsPP34	4	4.14	50.8
LOC_Os02g42270	OsPP35			
LOC_Os06g33530	OsPP86	5	15.22	64.7
LOC_Os06g33549	OsPP87			

a-distance between first and last gene in a group

b-Homology at amino acid level

### Expression profiles of OsPPs under abiotic stress conditions

**Figure 4:**
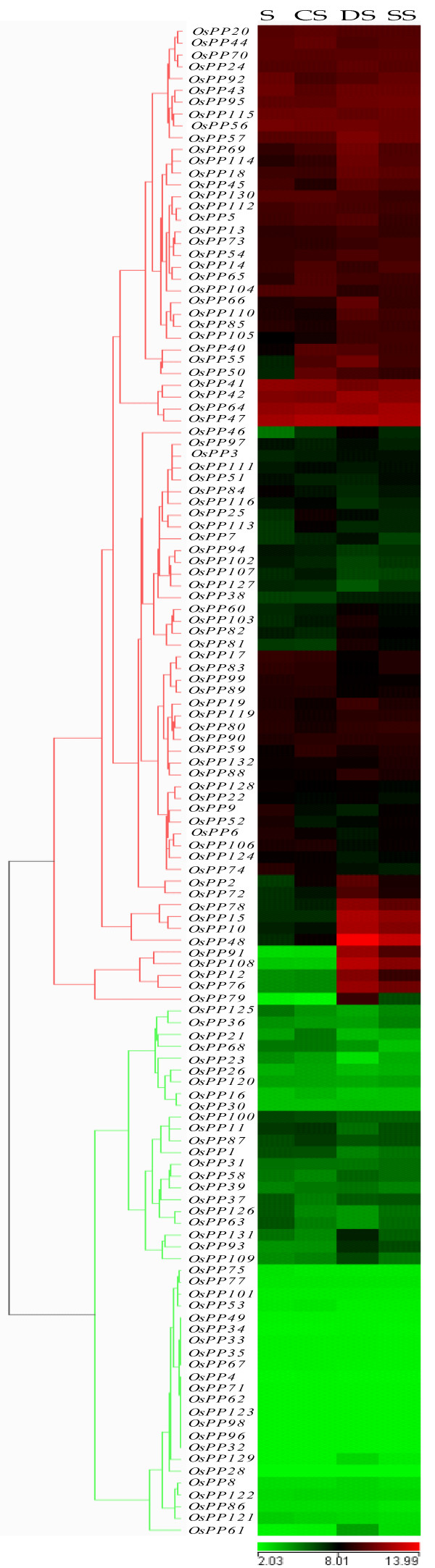
**Expression profiles of *OsPPs *under abiotic stress conditions**. Three experimental stress conditions are denoted as CS: Cold Stress, DS: Drought Stress, SS: Salt Stress and S: control, 7 days old unstressed seedling. Color bar at the base represents log2 expression values, thereby green color representing low level expression, black shows medium level expression and red signifies high level expression. A gene is considered differentially expressed under abiotic stress conditions if it is up- or down-regulated at least two-fold, at P-value ≤ 0.05, with respect to the 7-days-old unstressed seedling.

**Figure 5:**
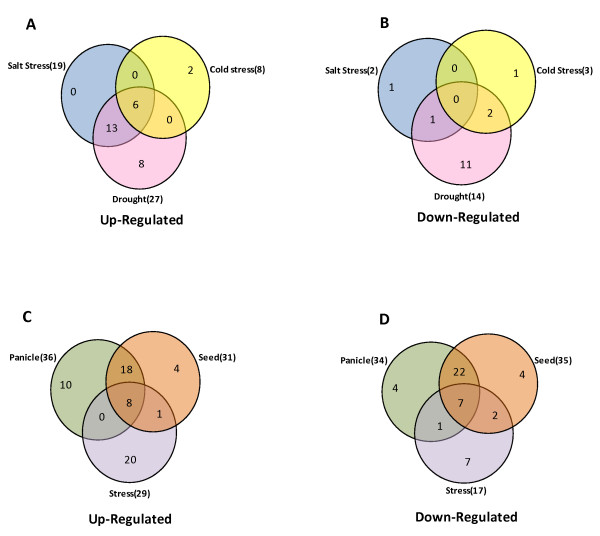
**Venn diagram for differentially expressed *OsPPs***. Protein phosphatase genes up-regulated **(A), **down-regulated **(B) **under different abiotic stress conditions. Different compartments showing the genes specific to either one particular stress (salt or drought or cold), involved in two stresses, or involved in all the three stresses. Protein phosphatase genes up-regulated **(C), **down-regulated **(D) **in stress and reproductive development showing overlapping expression pattern. Different compartments showing the genes specific to stress, panicle or seed stage or involved in stress-panicle, stress-seed or seed-panicle or involved in all the three conditions.

Expression profiles of *OsPPs *in 7-days-old seedlings were analyzed under three abiotic stress conditions (salt, cold and drought). After defining a criterion of fold change value > 2 (either up- or down-regulated) in comparison to untreated 7-days-old seedling control, a total of 46 *OsPP *genes were found to be differentially expressing (Figure 4). Out of these 46 genes, 31 were up-regulated and 15 were down-regulated in any of these above mentioned abiotic stresses. 6 *OsPP *genes (*OsPP2, OsPP40, OsPP46, OsPP48, OsPP50 *and *OsPP55*) were up-regulated whereas none of the genes was down-regulated in all the three stress conditions tested. We did not find any gene, which was up-regulated in both salt and cold or in both cold and drought stress together but 13 *OsPP *genes were up-regulated in salt and drought stress together. On the other hand, 1 and 2 genes were down-regulated in salt and drought or cold and drought stress together, respectively (Figure 5, Additional file 7). Observation for genes, expressing exclusively in any one of the three abiotic stresses identified 0, 2 and 8 genes getting up-regulated, whereas 1, 1 and 11 genes being down-regulated under salt, cold and drought stress, respectively (Figure 5a, b). MPSS expression data analysis revealed one more gene (*OsPP118*), which was not found on the Affymetrix gene chip, to be up-regulated under high salinity conditions (Additional file 8).

### Expression profiles of OsPPs during development

**Figure 6:**
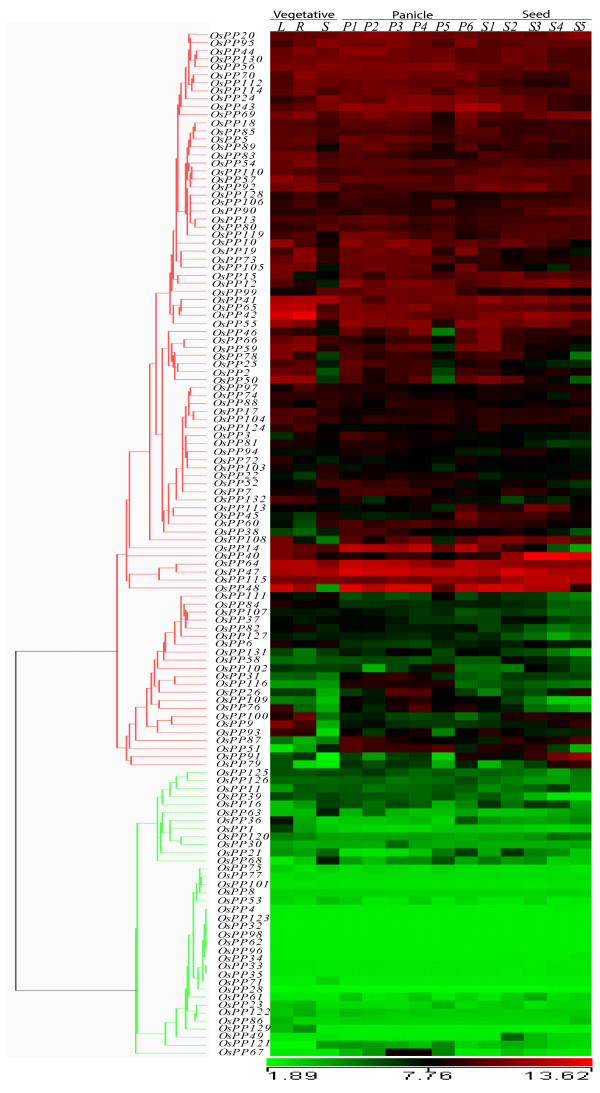
**Expression profiles of *OsPPs *during reproductive development**. Reproductive development comprising six stages of panicle [P1 (0-3 cm), P2 (3-5 cm), P3 (5-10 cm), P4 (10-15 cm), P5 (15-22 cm), and P6 (22-30 cm)] and five stages of seed [S1 (0-2 DAP), S2 (3-4 DAP), S3 (4-10 DAP), S4 (11-20 DAP) and S5 (21-29 DAP)] development. Genes are considered as up- or down-regulated w.r.t. all the vegetative controls, (L-mature leaf, R-root, and S-7-days-old seedling). Clustering of the expression profile was done with log transformed average values taking mature leaf as base line. The color scale at the bottom of the heat map is given in log2 intensity value. A gene is considered differentially expressed during reproductive development if it is up- or down-regulated at least two-fold, at P-value ≤ 0.05, with respect to the three vegetative controls (mature leaf, root and 7-days-old seedling).

Genome wide expression profiles for rice *OsPPs *genes during development were generated by analyzing microarray expression data obtained from Affymetrix rice whole genome arrays. Corresponding probe sets for 128 genes were found on Affymetrix gene chip; hence their expression profile could be analyzed. For expression analysis during reproductive development, 6 panicle stages (P1-P6) and 5 seed (S1-S5) development stages were compared with three combined vegetative developmental stages namely mature leaf, root and seedling (Figure 6). In total, 82 *OsPP *genes were found to be expressing differentially (with fold change > 2) during various developmental stages (Additional file 9). Out of these, 36 and 31 were up-regulated in panicle and seed tissues, respectively. Transcript levels for 18 *OsPPs *were commonly up-regulated in both the reproductive developmental phases. There were 10 and 4 genes, which were exclusively up-regulated during panicle and seed development, respectively. On the other hand 34 and 35 *OsPPs *were found to be down-regulated during panicle and seed development, respectively. 7 genes were commonly down-regulated in both panicle and seed stages together, whereas 4 genes each were exclusively down-regulated in panicles and seeds, separately (Figure 5c, d).

To understand the relationship between abiotic stresses and different developmental stages, we compared the expression profiles during various stages of reproductive development and under stresses. Among the genes expressing differentially both under abiotic stresses and during developmental stages, 8 were up-regulated whereas 7 were down-regulated together (Figure 5c, d). In addition, 4 genes were up-regulated in all the three abiotic stresses whereas they were down-regulated in most of the panicle development stages.

**Figure 7:**
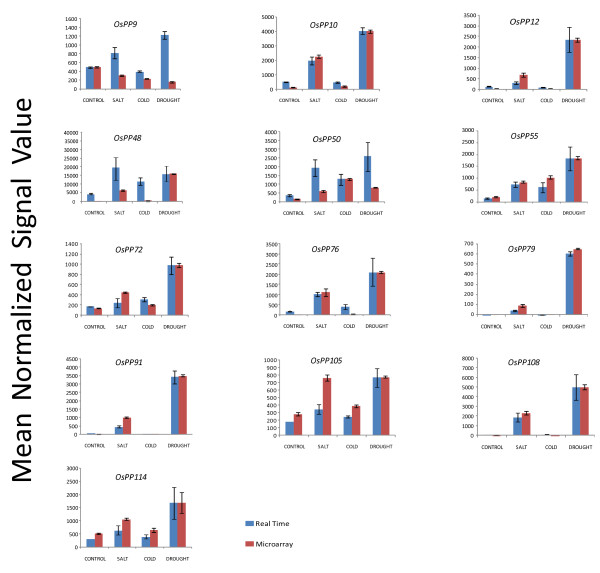
**Validation of expression profiles for selected *OsPPs *by Q-PCR**. Two and three biological replicates were taken for Q-PCR and microarray analysis respectively. Standard error bars have been shown for data obtained using both the techniques. Y-axis represents raw expression values obtained using microarray and Q-PCR expression values normalized with the maximum average value obtained by microarray data and X-axis shows different experimental conditions; red bars represent the expression from microarrays, while blue bars represent the real-time PCR values.

Among the *OsPP *genes that were found to express differentially under abiotic stresse conditions, 13 were validated experimentally using real time PCR. 10 *OsPP *genes out of 13 showed anticipated expression pattern, and could be correlated with microarray expression pattern. However, one of the genes, *OsPP9*, which was found to be down-regulated in microarray data, showed a contradictory expression pattern and was up-regulated in real time expression analysis. Moreover, two genes, *OsPP48 *and *OsPP50*, showed higher expression levels as determined by the real time PCR analysis when compared to microarray data (Figure 7).

### Expression profiles of duplicated OsPPs

**Figure 8:**
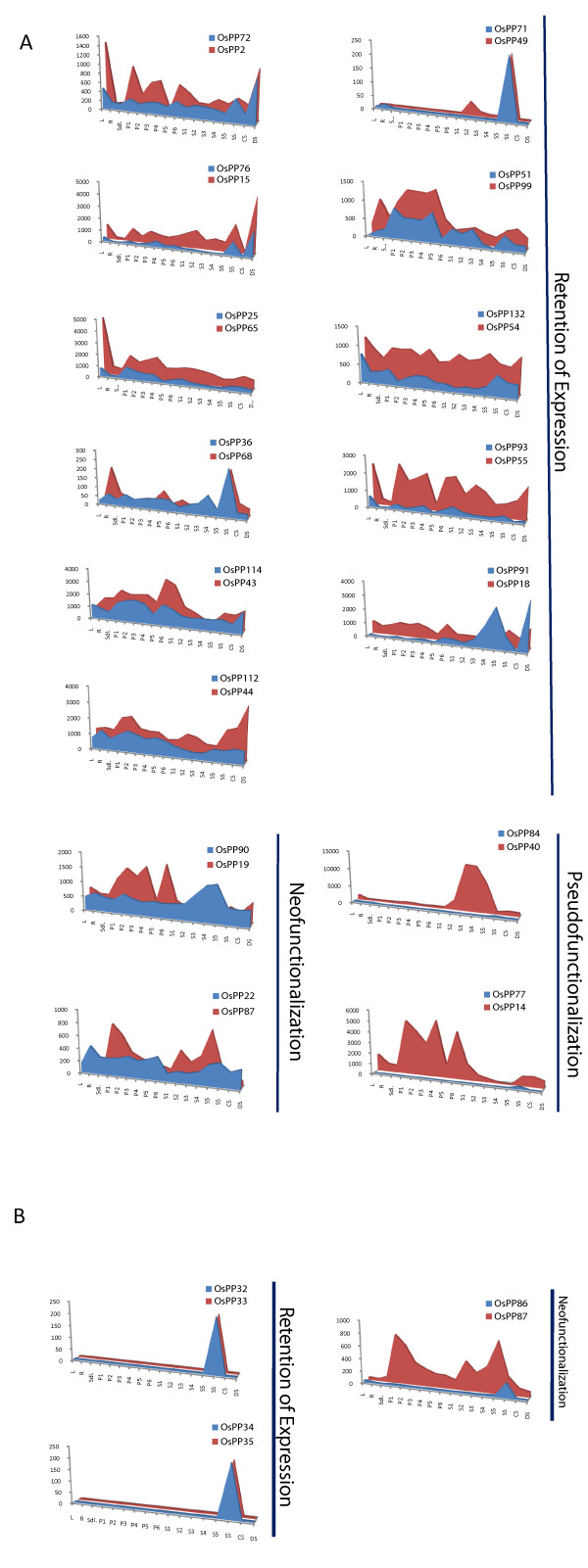
**Expression pattern of duplicated *OsPP *genes**. The expression values of duplicated genes obtained from microarray data were compared in leaf (L), root (R) and 7-day-old seedling (SDL) tissue, and in various stages of panicle development (P1-P6), seed development (S1-S5) and cold stress (CS), dehydration stress (DS) and salt stress (SS). Each area graph represents compilation of the mean normalized signal intensity values from 17 stages of development/stress conditions. Gene pairs have been grouped into retention of expression, neo-functionalization and pseudo-functionalization based on their respective profile **(A), **expression pattern of *OsPP *genes in segmentally duplicated region of rice genome and **(B), **expression pattern of *OsPP *genes in tandem duplication.

The expression pattern of *OsPP *genes present in segmentally duplicated regions and in tandem duplication was analyzed. Although, the entire duplicated gene pairs code for the catalytic subunit of protein phosphatases, varying expression pattern was observed. Out of the 20 pairs of segmentally duplicated genes, probe sets were available for 15 pairs on Affymetrix gene chip. The average signal values for all the samples (developmental as well abiotic stresses), are presented as an area-diagram (Figure 8). The expression pattern was very much similar for 11 pairs of genes indicating retention of function. However, the amplitude of expression varied in paired partners, which may be due to the fact that gene with low level of expression would tend to lose its function in due course of evolution. In two pairs (*OsPP14:OsPP77 *and *OsPP40:OsPP84*), one of the genes had almost negligible expression exhibiting pseudo-functionalization. For 2 pairs of gene (*OsPP19:OsPP90 *and *OsPP22:OsPP87*), expression pattern was very divergent for most of the tissue tested, indicating neo-functionalization. Expression analysis was also done for tandemly duplicated *OsPP *genes. From a total of 10 genes present in tandem duplication forming 5 groups, probe sets for only 3 pairs were available on Affymetrix gene chip. Two pairs of genes, *OsPP32:OsPP33 *and *OsPP34:OsPP35 *were having highly similar expression pattern and hence retention of expression, whereas, one pair *OsPP86:OsPP87 *showed divergent expression profile.

## Discussion

Protein phosphatases are a group of enzymes found ubiquitously in all prokaryotes and eukaryotes. This group of proteins is encoded by a large gene family in plants and is involved in the regulation of a number of cellular processes. This background knowledge prompted us to go for the identification of the full complement and expression profiling of this important gene family during development and under abiotic stresses. Based on keyword and HMM profile search in databases, we provide the evidence for the presence of 132 protein phosphatases coding genes in rice. Exploration for the full complement of protein phosphatases in *Arabidopsis *genome [25] resulted in the identification of 112 genes. Higher number of protein phosphatase genes in rice can be explained by the larger genome size (~389 Mb) as compared to *Arabidopsis *genome (~125 Mb). Also, the chromosomal duplication events might have resulted in the expansion of this gene family in rice. Based on domain search and phylogenetic analysis, we report the presence of 90 PP2C genes in rice representing largest phosphatase class, as already established in plants. Therefore, we are able to show a higher number of PP2C genes in rice than those given by a recent genome wide study (only 78 and 80 PP2C genes were reported in rice and *Arabidopsis*, respectively) [26]. Also, our dataset contains all the PP2C genes reported by them. In the present study, this higher number of PP2Cs in rice can be attributed to the genome wide search done using the HMM model. The large proportion of PP2C class in rice and *Arabidopsis *indicates the diverse role played by this gene family in plants. PP2A is another important class of ser/thr phosphatases and we could find 17 members belonging to this class in rice. Previously, 5 isoforms of catalytic subunit of PP2A have been reported in *Arabidopsis *[44-46]. As evident from previous study [47], we also could not find any gene belonging to PP2B class. Tyrosine phosphorylation is less common in plants as compared to ser/thr phosphorylations. In accordance with this observation, we could identify a single tyrosine specific phosphatase gene harboring PTP domain. Studies in *Arabidopsis *also identified only a single gene encoding the PTP [25,48]. Animals are known to have a large family of receptor tyr kinases, which interact with ligands at the plasma membrane and subsequently mediate tyr phosphorylation of large array of downstream targets. Plant genomes do not encode such receptor tyr kinases and hence, tyr phosphorylation in plants occurs less frequently than in animals [49,50]. We could also find several DSPs, which form another branch of protein tyrosine phosphatase class. In an earlier study, 22 DSPs were reported in *Arabidopsis *[51]. The number of protein tyrosine phosphatase genes in *Arabidopsis *and in rice is much lower than in humans, where more than 100 members of PTP superfamily, which including approximately 60 DSPs, have been reported [51]. Keeping in mind the fact that *Arabidopsis *has twice as many protein kinases than humans [52] and rice has even more [53], it is noteworthy that there is huge difference in the number of PTPs and DSPs between plants and humans. This implies that either the tyrosine phosphorylation components are limited or that the plant PTPs or DSPs could target many sites in the signaling processes.

During domain analysis, few other domains and motifs were found to be associated with main phosphatase domains, which included S_TKc (ser/thr kinase catalytic domain), FHA (forkhead associated domain), TPR (tetratricopeptide repeats) and EF-hand (calcium binding motif) (Additional file 5). These domains might be involved in Ca^2+ ^binding, structural organization or nuclear signaling. TPR (tetratricopeptide repeats) are the structural motifs found in a wide range of proteins. These mediate protein-to-protein interaction, thereby mediating the assembly of multi-protein complexes [54]. This type of domains has been found in a particular class (PP5) of protein phosphatases [55]. In PP5 phosphatases, these domains mediate the interaction with G proteins [56] and the small GTPase Rac protein [57]. FHA is a phosphoprotein-binding domain and has been found to be associated with a number of signaling proteins that interact with the partners, phosphorylated at serine/threonine residue. KAPP (kinase associated protein phosphatase) from *Arabidopsis *harbors this domain, where it has been found to play a crucial role in the interaction with RLKs (receptor like kinases) resulting in negative regulation of RLK signaling pathways, which are important for plant development [58]. During this analysis, we could find two PP2C genes (*OsPP58 *and *OsPP74*) with FHA domain, which turned out to be the kinase associated protein phosphatase (KAPP). The same genes were also found out as KAPP by one of the studies [59], with RGAP IDs, LOC_Os7g11010 and LOC_Os03g59530. This type of phosphatase gene has also been reported in *Arabidopsis *during the screening of a cDNA library for interaction with a RLK (receptor like protein kinase) protein kinase domain and has been finally characterized as the first downstream regulator of an RLK [16]. Phylogenetic analysis revealed close evolutionary relations among the members of the same class and some degree of divergence from members of other phosphatase classes. PP2Cs were found to be distributed into several sub-clades inside a major clade, which divide this family into various subfamilies. This is in accordance with the previous studies [26] and shows some degree of divergence even within the members of same class. The divergence might have resulted due to the presence of unique regulatory and targeting domains or by the attachment of regulatory subunits to the catalytic subunit of phosphatase [9]. To find out if phylogenetic relatedness could be correlated with functional conservations, as a first step their expression profiles were compared. Functionally, the phylogenetic structure explained that 6 genes (*OsPP10, OsPP12, OsPP48, OsPP76, OsPP79 *and *OsPP108*) with high expression values (up-regulated) under abiotic stresses were found to fall in the subfamily A of PP2C class and 2 genes (*OsPP40 *and *OsPP72*) in subfamily G. Two genes (*OsPP87 *and *OsPP91*) with higher expression values during the stages of panicle and seed development were found to fall in subfamily F2. On the other hand, all the genes from subfamily B were significantly down-regulated in most of the stages of panicle and seed development. This indicates that genes involved in similar functions have evolved from a common ancestor and are organized in closely related group. Moreover, the similar phylogenetic tree topologies of *Arabidopsis *and rice (Figure 2) suggest a common ancestry and evolutionary lineage for this gene family in two plant species from eudicots and monocots.

A number of *OsPP *genes were found to be duplicated either segmentally or in tandem, suggesting a role of chromosome gene duplication in the expansion and evolution of this gene family in rice. Duplicated *OsPPs *showed varying expression pattern during development and under abiotic stresses, which can be attributed to lack of intense selection pressure and need for diversification [60-63]. Segmentally duplicated genes are known to display a greater degree of functional divergence [61]. Consistent with this observation, duplicated genes in our study also exhibited pseudo-functionalization, neo-functionalization and retention of expression. Most of the segmentally duplicated gene pairs, retaining essentially similar expression profiles were found to have an amino acid level homology in the range of 62-94%. Therefore, we could correlate this high level of homology with the similarity of expression pattern in these gene pairs. The expression profiles were relatively less congruent in the case of tandemly duplicated genes, which is also evident from the low sequence similarity in the coding region and their respective regulatory sequences as well. Two of the segmentally duplicated gene pairs, *OsPP19:OsPP90 *and *OsPP22:OsPP87*, with relatively high levels of homology (83% and 56.7%, respectively), showed a complete divergent expression profile. This indicates that these genes might have undergone significant diversification after the duplication of the respective genomic segments, leading to neo-functionalization for the paired partners.

To find the probable explanation for this divergent expression pattern for duplicated genes, 1 kb upstream region from translation start site was explored. *In silico *promoter analysis revealed that 6 out of 11 segmentally duplicated gene pairs had 36-50% similarity in their regulatory elements (Additional file 10). The variability in the *cis*-acting regulatory elements of these genes might have resulted in the divergence in the amplitude of expression [64]. On the other hand, genes with striking differences in their expression pattern and those exhibiting neo-functionalization had only 14-21% similarity in their regulatory elements. It should also be kept in the mind that the eukaryotic genes with multiple introns and exons, apart from transcription level, are also regulated at the level of gene splicing. Many times alternative splicing leads to the generation of new protein isoforms and thus increases the genome complexity [65]. Plants have been shown to display a great variety in alternative splicing that is mainly of the intron retention type, whereas exon skip type is preferred in animals [66]. It has been shown that in rice 21.2% of the coding genome displayed alternative splicing [67] and its regulation by environmental stresses has been shown in *Arabidopsis *[68].

Keeping in view that the expression profile of a gene is the reflection of its functional relevance and provides a clue to get a deep insight into its functional role, genome-wide expression profiling of protein phosphatase gene family was carried out, using whole genome *indica *rice microarrays for vegetative, panicle and seed development stages; and three abiotic stress conditions (salt, cold and drought). In our analysis, a significant proportion of the *OsPPs *showed differential expression under various abiotic stresses and selected stages of panicle and seed development. The temporal and spatial display of gene expression might reflect the attainment of specialized functions by *OsPPs*. Our expression analysis revealed a differential as well overlapping pattern under abiotic stresses, such as cold, drought, and salinity. Earlier studies also suggest that the same gene can be activated by different triggers in a distinct signaling pathway [69,70]. This type of overlapping expression pattern among these genes might be the result of a common signaling component such as calcium, acting as "Hub" in the pathways triggered by different stress stimuli. This hub might act as a converging point for different stress signaling pathways and might activate the *cis*-acting regulatory elements of a respective gene under different abiotic stress conditions. Moreover, different pathways may share common components, which might be acting as "Node" and radiate towards more than one pathway or do crosstalk. Hence, may explain the overlapping expression patterns [71]. As it is well known that one of the earliest response to stress signals is manifestation of increased cellular calcium in plants [72,73], leading to activation of intermediate components such as calcium sensors including CaM (calmodulin), CBL (calcineurine B-like) and CDPK (calcium dependent protein kinases), which then modulate the activity of transcription factors, causing changes in gene expression. This hypothesis has been also supported by previous studies, where drought and cold treatments were shown to activate a single gene *RD29A *expression by activating the same *cis-*acting element, DRE/CRT [74]. However, different transcription factors like DREB1 and DREB2 were speculated to be involved in drought and cold responses, linking drought and cold pathways to *RD29A *expression [69,70]. In the light of these experimental evidences, it can be said that similar "Hub and Nodes" combination may be involved in stress signaling pathways constituting these phosphatases and the same *cis*-acting regulatory elements may be controlling the same gene in different signaling pathways, which could account for such an overlapping expression pattern. In our global gene expression analysis, where the entire spectrum of the reproductive development was analyzed by microarray based gene expression, we have been able to identify the genes relevant to the panicle and seed development. We could identify several *OsPPs*, which were commonly up- or down-regulated in both panicle and seed (at various developmental stages), whereas a subset of genes expressed differentially either in panicle or seed specifically. Since each reproductive stage analyzed in this study represented a complex set of tissues and cell types, the magnitude of the change in expression values of individual genes in a particular cell type may not be evident completely. Therefore, even a 2-fold estimated increase in the expression value could have high significance [41], as it would actually magnify several folds if only a particular cell-type or tissue was considered [74,75]. Here, the data shows that the genes up-regulated in narrow windows of reproductive development do not have very high expression signals, implying that their expression could be limited to specific cell types [76,77]. In our analysis, we have also attempted to figure out which genes have overlapping expression pattern in abiotic stresses and reproductive development, and interestingly found several genes commonly up- and down-regulated both under abiotic stresses and various stages of panicle and seed development. All the up-regulated genes belong to PP2C class whereas among the down-regulated ones, a subset of genes was from PP2C, PP2A and DSP classes. Such phosphatases with overlapping expression pattern were also reported by Yu et al. [78] where they showed that two PP2A catalytic subunit genes from rice, *OsPP2A-1 *and *OsPP2A-3*, had high expression level in stem and flower and low level in leaves [78]. Moreover, *OsPP2A-1 *was also highly expressed in roots but not *OsPP2A-3*. Transcript levels of *OsPP2A-1 *in roots and *OsPP2A-3 *in stems were found to be higher at the maturation and young stages, respectively. Expression level of both the genes was high in leaves subjected to drought and high salinity stress, whereas heat stress decreased the expression level of *OsPP2A-1 *in stems and induced *OsPP2A-3 *in all organs. These findings indicated that the two PP2Ac genes were subjected to developmental and stress-related regulation. This type of overlapping expression pattern in stress and developmental conditions can be attributed to some *cis*-acting regulatory elements, such as ABRE, which might be regulating both stress and development since desiccation is an integral part of both of these events. It is also well known that during the later stages of seed maturation, the developmentally programmed dehydration event is triggered leading to dormancy. Such dehydration events are mediated by phytohormone ABA, which also mediates drought and osmotic stress responses. Recently, it has been shown by triple mutant analysis that three SnRK2 protein kinases (SRK2D, SRK2E and SRK2I) are involved and essential in controlling the ABA mediated seed development in *Arabidopsis *[79]. Phosphatases such as ABI1 and ABI2, which halt ABA signaling, might also interfere in developmental processes, especially in the maturing phase of seed development, possibly by interacting with these SnRK2 protein kinases and blocking their signaling.

## Conclusions

Conclusively, this study presents a comprehensive account of protein phosphatase encoding genes and provides an insight into the phylogenetic relationship, organization, and gene duplications. Expression profiling of *OsPP *gene family has unraveled their probable functions during stress and development and has provided a platform to adopt genetic, physiological and molecular approaches for explication of the specific functions of candidate protein phosphatase genes in rice.

## Future prospects

Identification and expression profiling of whole complement of protein phosphatases in rice under abiotic stress and reproductive development has set the stage for answering several questions such as: What are the different stress signaling pathways in which various phosphatases are involved in rice? Whether particular types of phosphatases are confined to one particular signaling pathway or do they crosstalk with other signaling pathways? Whether the expression of a particular phosphatase is tissue and development specific or ubiquitous throughout the plant? Identification of developmentally regulated phosphatases may prompt studies involving promoter characterization. By utilizing such information, rice varieties can be generated, which can withstand and adapt to adverse environmental stresses like cold, drought and salinity at a particular developmental stage such as panicle formation and seed development.

## Authors' contributions

AS carried out computational analysis, microarray expression profiling, real time PCR validation of microarray data. JG was involved in computational and microarray data analysis. SK and AKT performed and analyzed the microarray expression data. GKP conceptualized, designed, and headed the project. AS and GKP wrote the manuscript. AS, SK, AKT and GKP participated in the revision of the final version of the manuscript. All authors read and approved the final manuscript.

## Supplementary Material

Additional file 1 **Table S1. Details of stress inducible rice genes used to verify the stress treatment**. Click here for file

Additional file 2 **Figure S1. Expression profile of reported stress inducible genes in rice**. A. Heat map showing stress inducible expression of some selected genes. Three experimental stress conditions are denoted as CS: Cold Stress, DS: Drought Stress, SS: Salt Stress and S: control, 7-days-old unstressed seedling. Color bar at the base represents baseline transformed values. B. Graph representing the differential expression pattern of selected stress inducible genes. X-axis denotes the RGAP database locus ID of the genes and Y-axis denotes fold change values w.r.t. to unstressed seedling (seedling baseline) Click here for file

Additional file 3 **Table S2. List of primers used for real time PCR expression analysis**. Click here for file

Additional file 4 **Figure S2. Domain organization of the protein phosphatase gene family in rice**. The SMART (http://smart.embl-heidelberg.de/) database was used to obtain the details of domain organization.10 major type of domain organizations include A. PP2Ac domain B. PP2Cc domain C. PP2C_SIG domain D. PTPc domain E. DSPc domain F. PTPc_DSPc domain G. LMWPc domain H. PP2Cc domain + Ser/thr kinase domain I. PP2Cc + FHA domain and J. PP2Ac + TPR domain. Click here for file

Additional file 5 **Table S3. Features of *OsPPs *in rice genome**. Click here for file

Additional file 6 **Figure S3. Phylogram depicting evolutionary relationship among the various phosphatase classes in rice**. A phylogram was made from the domain sequences of rice protein phosphatases. The phylogram was made in NJ Plot. PPs from rice were falling into different clades based on the bootstrap support value ≥ 50%. Click here for file

Additional file 7 **Table S4. Differential expression analysis of *OsPP *genes under abiotic stress conditions**. A gene is considered differentially expressed if it is up- or down-regulated at least 2 folds, at P value ≤ 0.05, with respect to 7 days old unstressed seedling. Click here for file

Additional file 8 **Table S5. MPSS data for 17 base signature**. Expression evidences from MPSS were obtained for all the *OsPPs*, which were not having corresponding probe set. Only those 17 base signatures, which uniquely identify the individual *OsPP*, were considered. The transcript abundance in parts per million (TPM) present in mRNA libraries is listed. Click here for file

Additional file 9 **Table S6. Differential expression analysis of *OsPP *genes during reproductive development**.
A gene is considered differentially expressed if it is up- or down-regulated at least 2 folds, at P value ≤ 0.05, with respect to all vegetative stages (seedling, mature leaf and root) Click here for file

Additional file 10 **Table S7. *cis-*regulatory elements analysis of duplicated genes using PlantCARE database**. Click here for file
