# Supplementary figures and images for: Protein phosphatase complement in rice: genome-wide identification and transcriptional analysis under abiotic stress conditions and reproductive development
Source: BMC Genomics. 2010 Jul 16;11:435. doi: 10.1186/1471-2164-11-435 (PMC3091634; doi:10.1186/1471-2164-11-435)

A

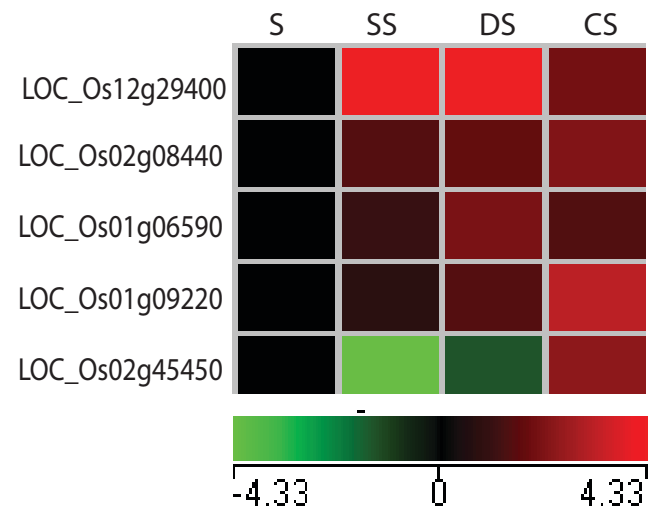

B

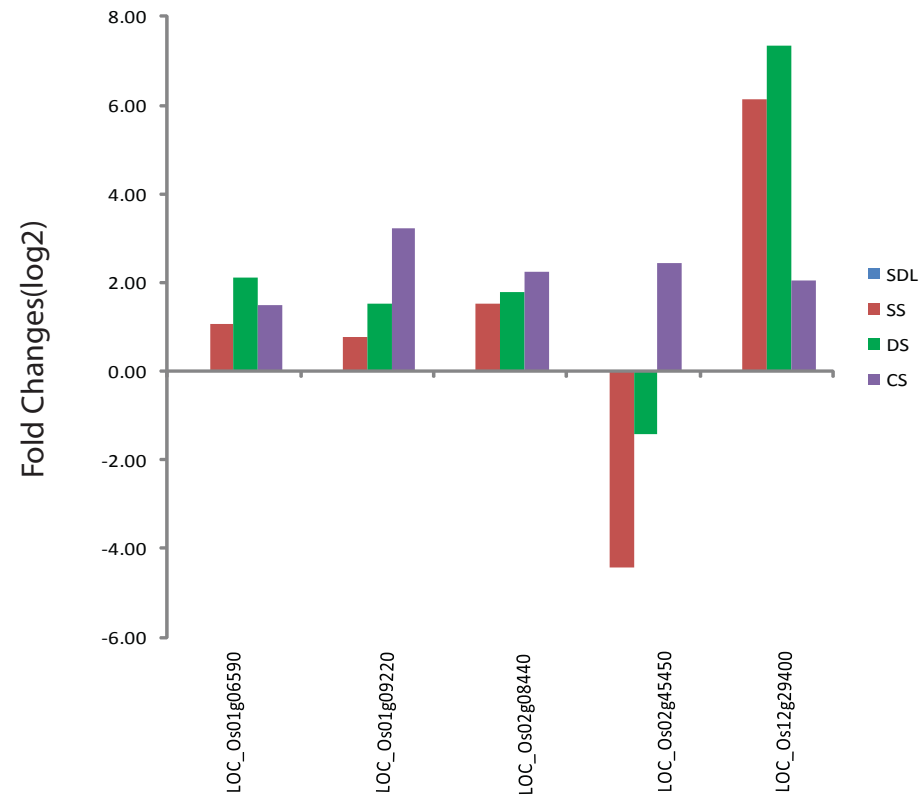

Supplement: Additional file 2 — Figure S1. Expression profile of reported stress inducible genes in rice. A. Heat map showing stress inducible expression of some selected genes. Three experimental stress conditions are denoted as CS: Cold Stress, DS: Drought Stress, SS: Salt Stress and S: control, 7-days-old unstressed seedling. Color bar at the base represents baseline transformed values. B. Graph representing the differential expression pattern of selected stress inducible genes. X-axis denotes the RGAP database locus ID of the genes and Y-axis denotes fold change values w.r.t. to unstressed seedling (seedling baseline) [file 1471-2164-11-435-S2.PDF]

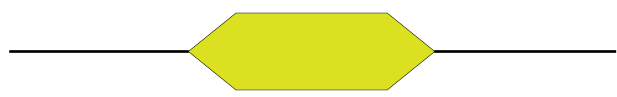

A

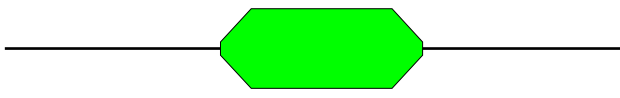

B

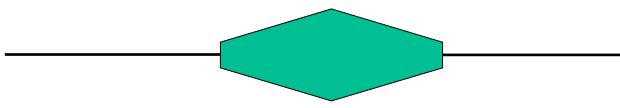

C

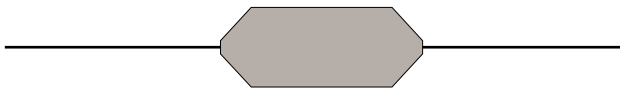

D

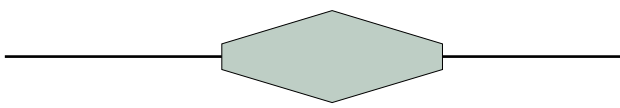

E

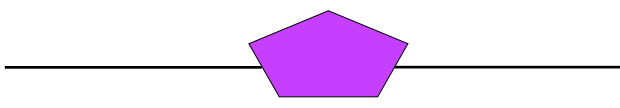

F

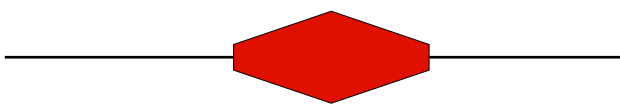

G

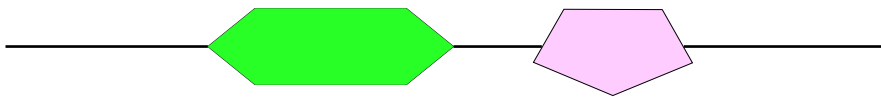

H

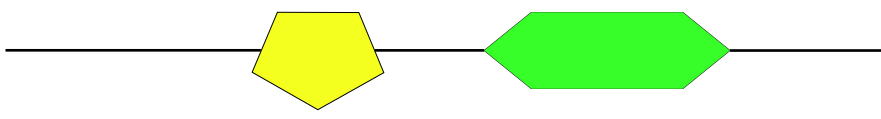

I

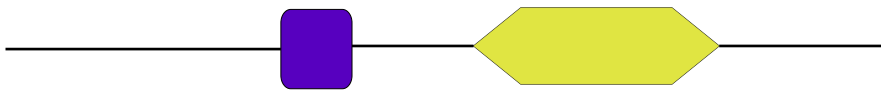

J

Supplement: Additional file 4 — Figure S2. Domain organization of the protein phosphatase gene family in rice. The SMART (http://smart.embl-heidelberg.de/) database was used to obtain the details of domain organization.10 major type of domain organizations include A. PP2Ac domain B. PP2Cc domain C. PP2C_SIG domain D. PTPc domain E. DSPc domain F. PTPc_DSPc domain G. LMWPc domain H. PP2Cc domain + Ser/thr kinase domain I. PP2Cc + FHA domain and J. PP2Ac + TPR domain. [file 1471-2164-11-435-S4.PDF]

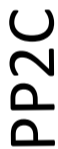

Supplement: Additional file 6 — Figure S3. Phylogram depicting evolutionary relationship among the various phosphatase classes in rice. A phylogram was made from the domain sequences of rice protein phosphatases. The phylogram was made in NJ Plot. PPs from rice were falling into different clades based on the bootstrap support value ≥ 50%. [file 1471-2164-11-435-S6.PDF]
